# Concurrent spontaneous pneumocephalus and pneumorrhachis due to polymicrobial infection: a case report

**DOI:** 10.3389/fmed.2026.1775521

**Published:** 2026-03-10

**Authors:** Shang Xiang, Daiping Hua, Liang Hong, Lanting Sun, Hongshuai Wu, Han Wang

**Affiliations:** 1 Department of Neurology, The First Affiliated Hospital of Anhui University of Chinese Medicine, Hefei, China; 2 Department of Neurology, The Fifth Affiliated Hospital of Anhui University of Chinese Medicine, Lu’an, China

**Keywords:** case report, intra-abdominal infection, pneumocephalus, pneumorrhachis, polymicrobial infection, suppurative encephalomyelitis

## Abstract

Intracranial pneumocephalus combined with intraspinal pneumorrhachis represents a scarce and highly lethal condition. We report the case of a 69-year-old woman who initially presented with status epilepticus, followed by progressive deterioration of consciousness. Imaging studies revealed tension pneumocephalus with concurrent intraspinal pneumorrhachis. Abdominal computed tomography (CT) demonstrated intestinal obstruction and gastrointestinal perforation. Although blood cultures yielded negative results, cerebrospinal fluid culture confirmed polymicrobial infection, suggesting purulent meningoencephalomyelitis secondary to intra-abdominal infection. The patient exhibited a complex clinical presentation with a rapidly progressive course. Despite aggressive treatment, she developed bilateral cerebellar hemispheric infarction and cerebral herniation, ultimately succumbing to the disease. This case is particularly noteworthy due to the unusual pathway of infection from the abdominal cavity to the central nervous system (CNS), accompanied by the distinctive radiological findings of both intracranial and intraspinal air accumulation. This report emphasizes the importance of clinical awareness and vigilance regarding such rare but life-threatening.

## Introduction

Pneumocephalus refers to the pathological presence of air within the intracranial cavity. Air may accumulate in the extradural, subdural, subarachnoid, intraventricular spaces, or within the brain parenchyma itself. Common etiologies include head trauma, neurosurgical procedures, paranasal sinus infections, and iatrogenic interventions (1, 2). Spontaneous pneumocephalus is considerably rare and predominantly results from microbial infections. Tension pneumocephalus represents the most critical form, with diverse clinical manifestations ranging from severe agitation and decreased consciousness to focal neurological deficits and even cardiac arrest, carrying an extremely high mortality rate (3, 4). Intraspinal pneumorrhachis is even more uncommon, with limited reports in the literature (5, 6). The concurrent presentation of pneumocephalus and intraspinal pneumorrhachis observed in this patient constitutes an exceptionally rare radiological finding in clinical practice. As of the present date, there have been no documented cases in the global literature of concurrent pneumocephalus and pneumorrhachis, nor have there been reports of polymicrobial CNS infections originating from intra-abdominal sources. Through detailed analysis of the clinical features, imaging characteristics, and therapeutic management of this case, we aim to provide valuable insights for early recognition and intervention in similar cases.

## Case presentations

A 69-year-old woman presented to our Department of Neurology on December 3, 2025, with a one-day history of limb rigidity, convulsions, and altered mental status. The previous day, she exhibited incoherent speech and inappropriate responses. On the morning of admission, she suddenly developed generalized limb rigidity with bilateral upper limb jerking and trismus, progressing to unresponsiveness and inability to eat or speak. These symptoms persisted, prompting her presentation to our emergency department, where she was admitted with a working diagnosis of status epilepticus. Throughout the hospital course, consciousness remained impaired with ongoing seizure activity. She remained afebrile with preserved spontaneous urination but experienced constipation. Body weight had been stable in recent weeks.

The patient had undergone surgery for endometrial cancer 10 years prior, followed by adjuvant radiotherapy and chemotherapy. She had a history of recurrent intestinal obstruction, which was previously managed at our Department of Gastrointestinal Surgery with clinical improvement. She had been maintained on a liquid diet since then. There was no history of hypertension, diabetes mellitus, hepatitis, tuberculosis, trauma, blood transfusion, or drug allergies. The patient was a retired rural resident from Lu’an, Anhui Province, supported by subsistence farming and of moderate socioeconomic status. She had no history of occupational exposure to hazardous substances or travel to endemic regions, nor any documented chronic infectious diseases. Lu’an is not endemic for any specific bacterial, viral, or vector-borne diseases, and no relevant infectious disease outbreaks were reported locally during her illness.

Upon admission, the patient’s vital signs were stable. She was stuporous and required a wheelchair for transport. The examination was limited by her poor cooperation; she appeared normally developed but poorly nourished (cachectic). Notably, abdominal palpation revealed positive signs of peritoneal irritation. Neurological examination showed bilateral pupils (3 mm) that were equal, round, and reactive to light, with no nystagmus observed. Nasolabial folds were symmetrical. Assessment of tongue protrusion and muscle strength was precluded by her clinical state; however, generalized hypertonia was noted, which was more pronounced in the upper extremities. Deep tendon reflexes were normal, and Babinski signs were negative bilaterally. Sensory and coordination testing could not be performed. Notably, meningeal irritation signs were positive.

**Figure 1:**
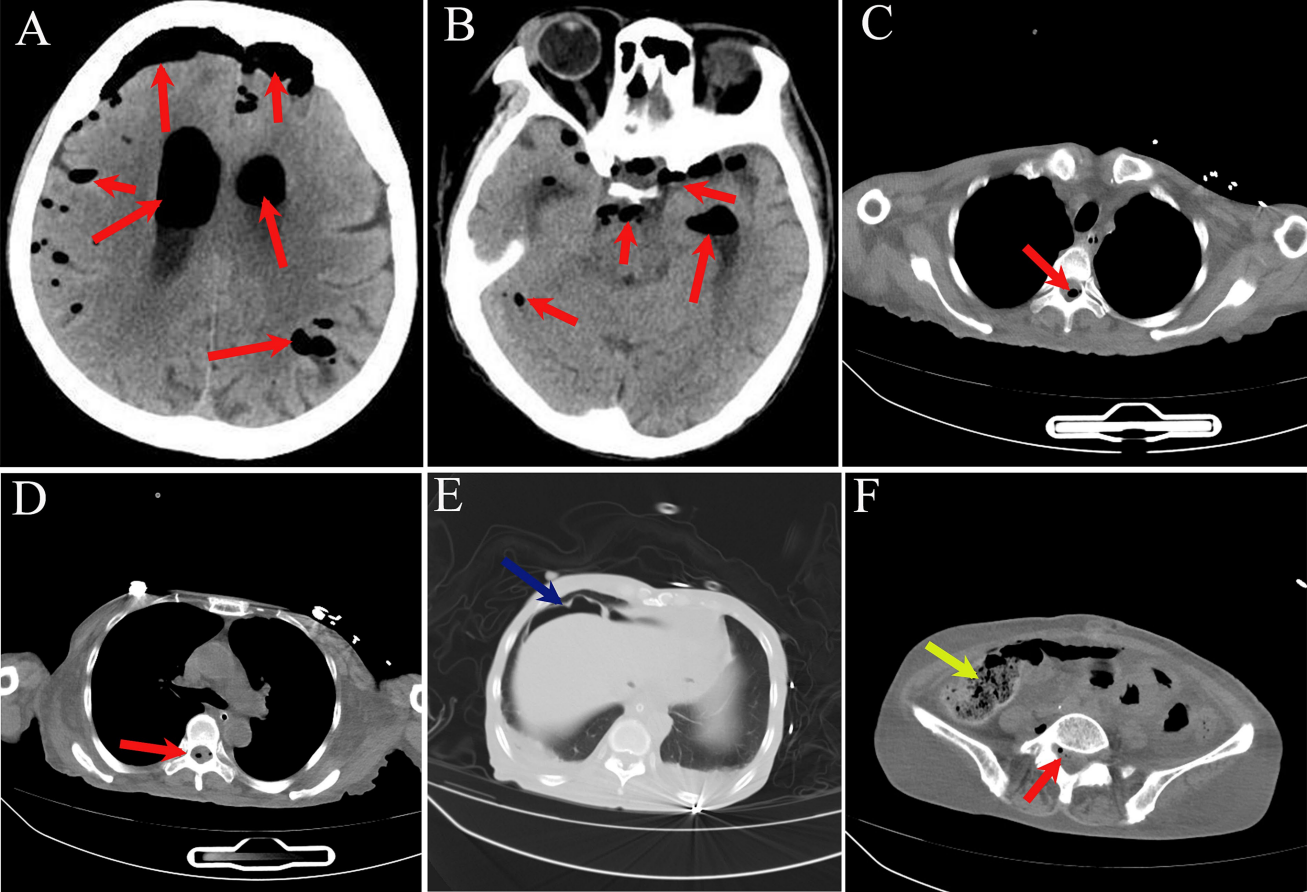
**(A–F)** CT of the head, thorax, and abdomen. Red arrows indicate pneumocephalus involving the intracranial space, ventricular system, and spinal canal; green arrows indicate free intraperitoneal air; yellow arrows indicate hyperdense fecalith.

Initial laboratory investigations revealed a white blood cell count of 11.18 × 10^9^/L. Inflammatory markers were markedly elevated, with a C-reactive protein (CRP) level of 355.98 mg/L and procalcitonin (PCT) of 2.45 ng/mL. Coagulation profiles were deranged, showing a prolonged prothrombin time (PT) of 48.58 s and an elevated D-dimer of 5.44 μg/mL. Biochemical analysis was significant for severe hypokalemia (2.61 mmol/L) and hypoalbuminemia (24.10 g/L; total protein: 55.30 g/L), while transaminase levels and renal function remained within normal limits. Urinalysis was indicative of infection, with a bacterial count of 1113.10/μL. CT of the head, thorax, and abdomen demonstrated critical findings: extensive pneumocephalus within the intracranial compartments and ventricular system, accompanied by pneumorrhachis. Thoracic imaging showed minor bilateral pleural effusions. Abdominal and pelvic CT revealed evidence of hollow organ perforation, characterized by pneumoperitoneum and diffuse bowel distension. Furthermore, multiple high-density fecaliths were noted within the colorectum, associated with significant abdominopelvic ascites (Figure 1).

**Figure 2:**
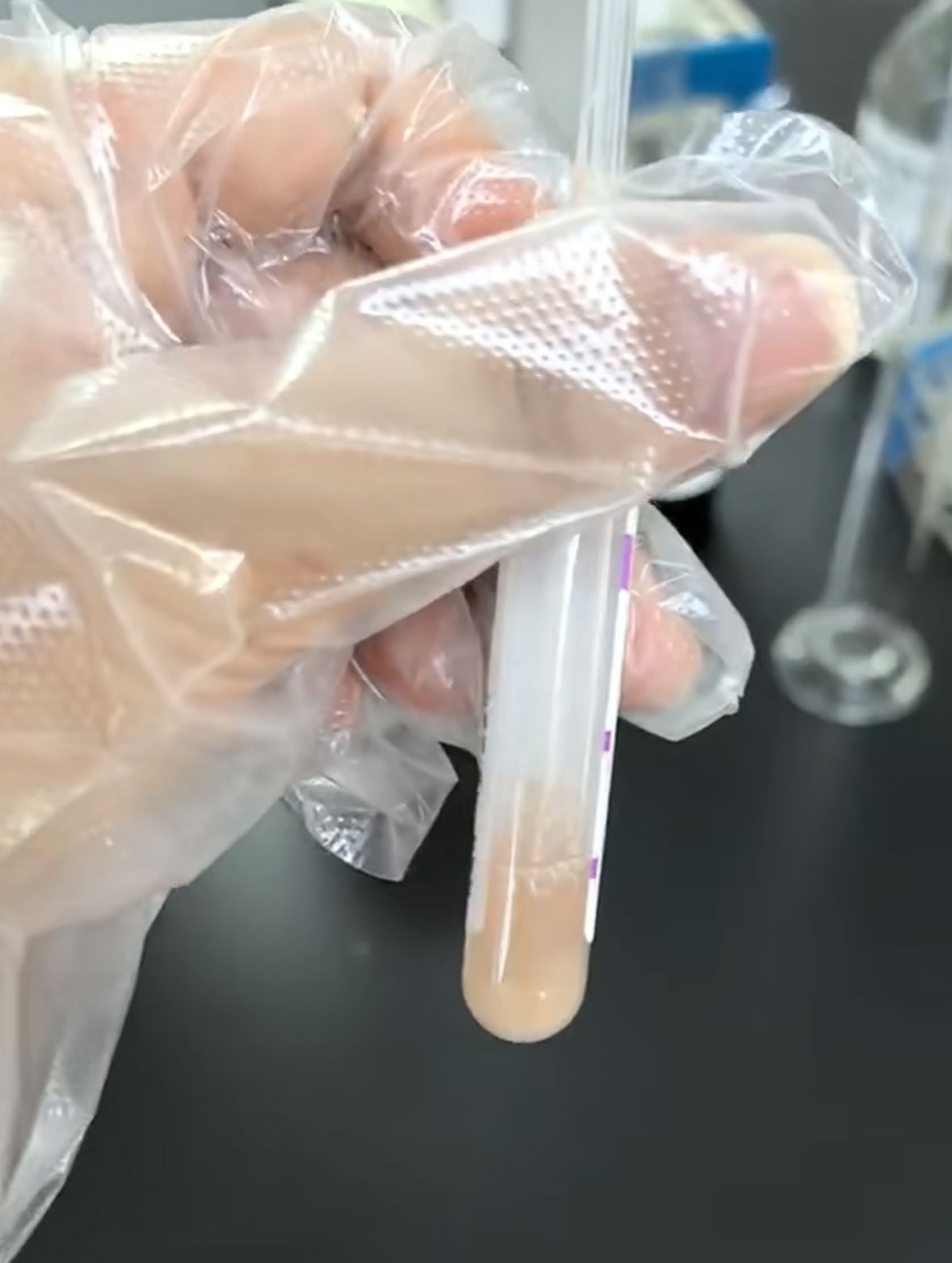
Cerebrospinal fluid.

Clinical management and further investigations: based on the clinical presentation, empirical broad-spectrum antimicrobial therapy was promptly initiated, targeting both Gram-positive and Gram-negative pathogens. Comprehensive supportive treatments were simultaneously administered, including the correction of coagulopathy, intravenous fluid resuscitation, pharmacological seizure control, and replenishment of electrolytes. Given the high clinical suspicion of a CNS infection supported by systemic inflammatory markers, a lumbar puncture was performed for definitive diagnosis. The procedure revealed a significantly elevated opening pressure of 360 mmH_2_O. The cerebrospinal fluid (CSF) appeared grossly milky white and purulent (Figure 2). Laboratory analysis of the CSF demonstrated a markedly elevated white blood cell count of 75,938 × 10^6^/L, a severely diminished glucose level of 0.49 mmol/L, chloride of 112.45 mmol/L, adenosine deaminase (ADA) of 58.70 U/L, and a significantly high protein concentration of 6,014.00 mg/L. These CSF findings, in conjunction with the radiological evidence of gas within the spinal canal and cranium, confirmed a diagnosis of pyogenic encephalomyelitis. To further investigate the etiology of the pneumocephalus, a high-resolution 3D reconstruction CT of the skull base was performed, which effectively ruled out any traumatic skull base fractures.

**Figure 3:**
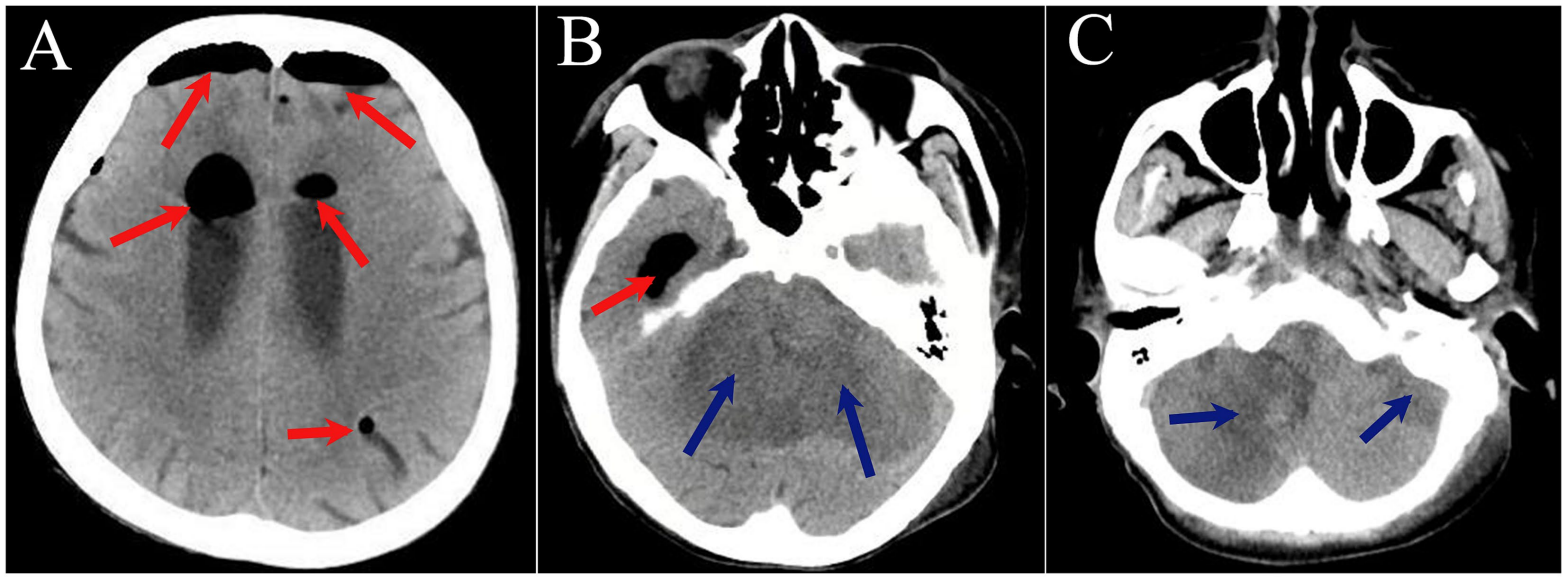
**(A–C)** CT of the head. Red arrows indicate pneumocephalus involving the intracranial space and ventricular system; green arrows indicate hypodense lesions in bilateral cerebellar hemispheres.

**Figure 4:**
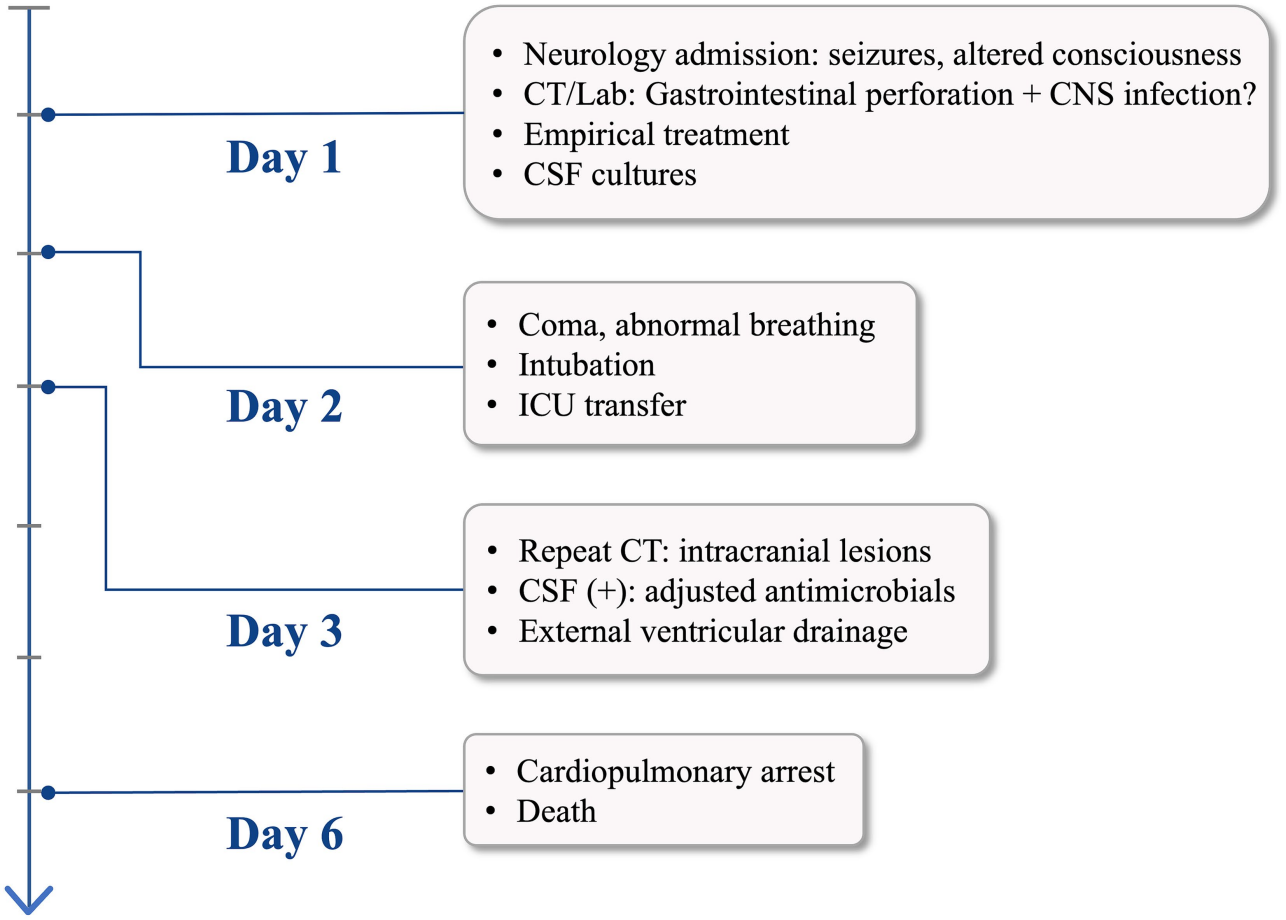
Patient clinical course timeline.

On hospital day 2, the patient’s condition deteriorated with loss of consciousness and abnormal respiratory pattern. Endotracheal intubation was performed with mechanical ventilation, and she was transferred to the intensive care unit for further management. Repeat head CT on hospital day 3 revealed tension pneumocephalus, bilateral cerebellar hemispheric infarction with compression of the fourth ventricle and ambient cistern, and cerebral herniation (Figure 3). To decompress the tension pneumocephalus and reduce intracranial pressure, right lateral ventricular drainage was performed under local anesthesia on the same day, the intraventricular pressure was measured as 410 mmH₂O. Blood cultures remained negative for both aerobic and anaerobic organisms. However, CSF cultures yielded *Enterococcus faecium, Escherichia coli, and Candida albicans,* with antimicrobial susceptibility testing indicating multidrug resistance. Based on the patient’s clinical history and comprehensive diagnostic workup, the final diagnosis was purulent meningomyelitis with tension pneumocephalus, diffuse peritonitis, coagulopathy, and electrolyte imbalance. Despite intensive treatment, the patient developed cardiopulmonary arrest on hospital day 6 and expired despite resuscitative efforts. The clinical timeline is detailed in Figure 4.

## Discussion

Pneumocephalus accompanied by intraspinal pneumorrhachis remains exceedingly rare in clinical practice. Pneumocephalus can arise from various etiologies, including skull base fractures, surgical procedures, iatrogenic causes, and microbial infections (7). Infection-related cases are predominantly attributed to pulmonary, otogenic, or sinonasal sources (8, 9). Central nervous system infection secondary to intra-abdominal pathology, resulting in pneumocephalus with concurrent intraspinal air, has seldom been documented. We report a 69-year-old woman who presented with status epilepticus and rapidly progressed to altered consciousness, ultimately succumbing to bilateral cerebellar infarction and herniation. The most striking features of this case were the radiographic demonstration of tension pneumocephalus with intraspinal air and the microbiological confirmation of polymicrobial infection involving *Enterococcus faecium, Escherichia coli,* and *Candida albicans* on CSF culture. This unusual presentation underscores the necessity for clinicians to maintain a comprehensive approach when evaluating central nervous system infections. Potential sources throughout the body, particularly occult intra-abdominal foci, warrant careful consideration to avoid diagnostic oversight.

The patient’s medical history of endometrial surgery, chemoradiotherapy, and recurrent bowel obstruction represented critical predisposing factors. Pelvic radiation induces intestinal fibrosis, compromises vascular supply, and increases bowel wall fragility, predisposing to perforation. Additionally, radiation disrupts the gut microbiome, facilitates overgrowth of opportunistic pathogens, and impairs both local and systemic immunity (10). Recurrent bowel obstruction causes sustained elevation of intraluminal pressure, exacerbating intestinal wall injury and promoting bacterial translocation with subsequent toxin release. Prolonged liquid diet resulted in malnutrition, evidenced by a serum albumin of only 24.10 g/L on admission, reflecting severe immunocompromise that created a favorable milieu for infection. Abdominal CT demonstrated free intraperitoneal air, distended bowel loops, and abdominopelvic fluid collection, confirming bowel obstruction with gastrointestinal perforation. This provided the anatomical substrate for intra-abdominal sepsis. CSF cultures yielded polymicrobial infection with multidrug-resistant organisms. The identification of *Enterococcus faecium and Escherichia coli*, typical gut commensals, pointed to an abdominal origin (11, 12). The characteristic purulent CSF findings (milky appearance, WBC count 75,938 × 10^6^/L, profound hypoglycorrhachia, and markedly elevated protein) confirmed purulent meningomyelitis. Notably, blood cultures were negative, though this does not exclude hematogenous spread. Two potential routes of CNS infection warrant consideration: first, hematogenous dissemination from the abdominal focus; second, direct ascent of intra-abdominal pathogens along the subarachnoid space surrounding lumbosacral nerve roots, facilitating invasion into the central nervous system and culminating in purulent meningitis and myelitis.

*Escherichia coli* represents the predominant bacterial species in the intestinal tract of humans and many animals. It possesses multiple virulence factors, including endotoxin, capsule, adhesins, and exotoxins. This organism ferments various carbohydrates to produce acid and gas. While *Escherichia coli* is a normal commensal, it can cause both enteric and extraintestinal diseases under certain conditions. Central nervous system infections caused by *Escherichia coli* occur predominantly in infants, though adult cases have been reported (13, 14). *Enterococcus faecium* exhibits lower pathogenicity; infection in this patient was closely related to her underlying conditions and gastrointestinal perforation following bowel obstruction (15). Although *Candida albicans* is a common opportunistic pathogen, isolated chronic meningitis is exceptionally rare, typically arising from hematogenous dissemination during candidemia (16, 17). The polymicrobial infection identified in CSF likely led to extensive necrosis and liquefaction of brain and spinal cord tissue in the setting of purulent meningomyelitis. Necrotic tissue provided an optimal substrate for microbial proliferation with substantial gas production. Gas accumulated within the cranial and spinal cavities, and because of poor absorption coupled with ongoing production, tension pneumocephalus developed. Tension pneumocephalus causes rapid intracranial pressure elevation, compressing the brainstem and cerebellum, resulting in altered consciousness, abnormal respiratory patterns, and ultimately herniation. This patient deteriorated from confusion to coma within 24 h of admission. Repeat CT showed bilateral cerebellar infarction, likely attributable to basilar artery compression (18). Intraspinal air formation similarly resulted from spinal cord parenchymal necrosis in purulent myelitis, with gas disseminating through microscopic channels.

Despite rapid diagnostic confirmation, comprehensive medical management, and ventricular drainage to decompress elevated intracranial pressure, the multidrug-resistant pathogens proved refractory to antimicrobial therapy. The infection advanced unchecked throughout the central nervous system, resulting in fatal herniation. This study has several notable limitations that merit acknowledgment. As a single-case report, its findings lack generalizability and cannot be extrapolated to larger or more diverse patient cohorts. Furthermore, the absence of postmortem autopsy data precludes definitive confirmation of the precise anatomical pathway mediating the invasion of intra-abdominal pathogens into the central nervous system.

## Conclusion

This case is remarkable not only for the unusual radiographic findings of concurrent intracranial and intraspinal pneumorrhachis but also for the complex pathophysiological cascade it demonstrates: intra-abdominal infection extending to the central nervous system, causing purulent meningomyelitis with subsequent tissue necrosis and gas production, evolving into tension pneumocephalus and intraspinal air accumulation, ultimately progressing to fatal herniation from intracranial hypertension. This case underscores the importance of considering extra-cerebral sources when evaluating CNS infections of unclear etiology. Clinicians should look beyond focal intracranial pathology, particularly in patients with abdominal disease history, and maintain awareness of potential abdomen-to-CNS transmission routes. Diagnostic workup should prioritize assessment for intra-abdominal infectious foci alongside neuroimaging. Early synchronous radiological screening of the CNS and abdomen, combined with timely microbiological sampling, can facilitate prompt identification of occult extracranial infection sources and CNS involvement. Multidisciplinary collaboration may optimize source control and intervention, potentially improving outcomes in such rare and life-threatening cases.

## Patient perspective

The patient’s daughter stated: “My mother’s health declined after endometrial cancer surgery 10 years ago, and she was repeatedly hospitalized for bowel obstruction, surviving on a liquid diet. Her condition worsened extremely rapidly this time. Despite prompt diagnosis and aggressive treatment, her baseline health was too poor to overcome the severe infection.”

## Data Availability

The original contributions presented in the study are included in the article/supplementary material, further inquiries can be directed to the corresponding author.
